# The impact of bone marrow fibrosis and JAK2 expression on clinical outcomes in patients with newly diagnosed multiple myeloma treated with immunomodulatory agents and/or proteasome inhibitors

**DOI:** 10.1002/cam4.3265

**Published:** 2020-07-06

**Authors:** Barry Paul, Yue Zhao, Gavin Loitsch, Daniel Feinberg, Parker Mathews, Ian Barak, Megan Dupuis, Zhiguo Li, Lindsay Rein, Endi Wang, Yubin Kang

**Affiliations:** ^1^ Division of Hematologic Malignancies and Cellular Therapy Duke University Medical Center Durham NC USA; ^2^ Department of Pathology Duke University Medical Center Durham NC USA; ^3^ Biostatistics Shared Resource, Duke Cancer Institute Duke University Medical Center Durham NC USA; ^4^ Hematology/Oncology Fellowship Program MD Anderson Cancer Center, University of Texas Houston TX USA

**Keywords:** bone marrow fibrosis, immunohistochemical staining, JAK2, multiple myeloma, overall survival, progression free survival

## Abstract

We determined the impact of bone marrow fibrosis (BMF) on the clinical outcomes of newly diagnosed multiple myeloma (NDMM) patients in the current era of myeloma therapy. A total of 393 MM patients were included in the final analysis. The median followup was 83 months (range: 3.9 to 212 months). BMF was noted in 122 (48.2%) evaluable patients. Median progression free survival (PFS) in patients without BMF was 30.2 (95% CI: 24.7‐38.0) months, and 21.1 (95% CI: 18.8‐27.5) months in patients with BMF present (*P* = .024). Median overall survival (OS) was 61.2 (95% CI: 51.5‐81.2) months in patients without BMF, and 45.1 (95% CI: 38.7‐57.0) months in patients with BMF (*P* = .0048). A subset of 99 patients had their bone marrow biopsies stained for JAK1 and JAK2 by immunohistochemistry. Of these samples 67 (67.7%) patients had detectable JAK2 expression predominantly noted on bone marrow megakaryocytes. JAK2 expression correlated with myeloma disease stage (*P* = .0071). Our study represents the largest dataset to date examining the association of BMF with prognosis in the era of novel therapies and widespread use of hematopoietic stem cell transplant (HSCT). Our data suggest that MM patients with BMF (particularly those with extensive BMF) have a poorer prognosis even when treated with immunomodulatory agents and proteasome inhibitors.

## Graphical abstract

We determined the impact of bone marrow fibrosis (BMF) on the clinical outcomes of newly diagnosed multiple myeloma patients in the current era of myeloma therapy. We found that BMF is common in myeloma and MM patients with BMF (particularly those with extensive BMF) have a poorer prognosis even when treated with immunomodulatory agents and proteasome inhibitors.

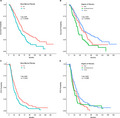

## 1 INTRODUCTION

Multiple Myeloma (MM) is a malignancy of terminally differentiated plasma cells and accounts for about 1% of all cancers and 10% of all hematological malignancies in the United States.^1^ The management of newly diagnosed multiple myeloma (NDMM) has evolved significantly over the last two decades. In 2005, the international staging system (ISS) was reported and has since been utilized globally for MM classification and stratification.^2^ In 2009, the International Myeloma Workshop developed guidelines to incorporate metaphase karyotype and fluorescence in situ hybridization (FISH) results for myeloma cytogenetic risk stratification and prognosis.^3^, ^4^ Within the past 15 years several novel therapies such as the immunomodulatory agents (IMiDs, ie, thalidomide, lenalidomide, and pomalidomide), the proteasome inhibitors (PIs, ie, bortezomib, carfilzomib, and ixazomib), and the monoclonal antibodies (ie, daratumumab, isatuximab, and elotuzumab) have been approved for the treatment of MM and now are widely incorporated in the treatment of myeloma patients. The three drug combination of lenalidomide, bortezomib, and dexamethasone as induction therapy, followed by autologous hematopoietic stem cell transplant (HSCT) has resulted in a response rates of over 90% and a median overall survival (OS) of 7‐9 years.^5^, ^6^, ^7^, ^8^

Despite these improvements, MM remains an incurable disease, and nearly all myeloma patients will eventually relapse. Recently, the bone marrow (BM) microenvironment has been shown to play an important role in the survival and clonal evoluation of myeloma cells as well as the development of drug resistance.^9^, ^10^, ^11^, ^12^, ^13^ The BM microenvironment is complex and is composed of extracellular matrix proteins, cytokines/chemokines, BM stromal cells, mesenchymal stem cells, osteoblasts and osteoclasts, inflammatory cells, megakaryocytes, and microvessels.^14^ Understanding the alterations in the BM microenvironment and the molecular pathways related to these changes are crucial to further improving the efficacy of myeloma treatment and the outcomes of patients with MM.

Bone marrow fibrosis (BMF) is the deposition of reticulin or collagen in the BM stromal environment. Reticulin is a normal component of the BM microenvironment and can be increased in a wide variety of malignant and nonmalignant diseases. There have been several case reports and small series studies documenting the association of BMF and plasma cell dyscrasias including MM,^15^, ^16^, ^17^, ^18^, ^19^, ^20^, ^21^, ^22^ with the frequency of BMF being 8%‐57%.^23^, ^24^ These studies suggested that presence of BMF was related to the magnitude of plasma cell infiltration and was associated with poorer prognosis in MM patients.^17^, ^18^ However, the majority of these studies were conducted prior to 2000 when low doses of melphalan and prednisone were still the mainstay of MM treatment, and prior to the advent of cytogenetic risk stratification and ISS staging.

The objectives of this study were: (a) to determine the incidence, patient characteristics, and clinical outcomes of myeloma patients with BMF in the current era of treatment; (b) to determine the correlations between BMF and ISS stage, and cytogenetic risk stratification; and (c) to determine the roles/contributions of Janus Kinase (JAK)1 and JAK2 in myeloma patients with BMF.

## 2 METHODS

### 2.1 Approval

The study was approved by the Institutional Review Board (IRB) at Duke University Medical Center and was conducted in accordance with the Declaration of Helsinki and the Health Insurance Portability and Accountability Act guidelines of 1996.

### 2.2 Retrospective chart review

A single center, retrospective cohort study was performed. Patients were included in the study if they met the following criteria: pathologic confirmation of NDMM diagnosis by Duke review, seen at the Duke University MM clinic between 2003 and 2013, BM biopsy performed at the time of diagnosis or within 12 months of starting treatment for MM, and medical records available that included laboratory data at the time of diagnosis, treatment regimen, and survival status.

Patient data were collected from our database and by review of the patients’ electronic medical records. The treatment response was characterized using the International Myeloma working group (IMWG) treatment response criteria and classified as complete remission, very good partial response, partial response, stable disease, or progressive disease.^4^, ^25^ The ISS stage and the cytogenetic risk were defined using IMWG criteria.^2^, ^3^, ^4^ Progression free survival (PFS) was defined as the duration from the initiation of treatment to first progression or death, whichever was earlier. OS was defined as the duration from the date of diagnosis of MM to the date of death or date of last follow‐up at which patient was known to be alive, with those alive censored at the date of last contact.

### 2.3 Determination of bone marrow fibrosis and CD138 + myeloma cells

**FIGURE 1:**
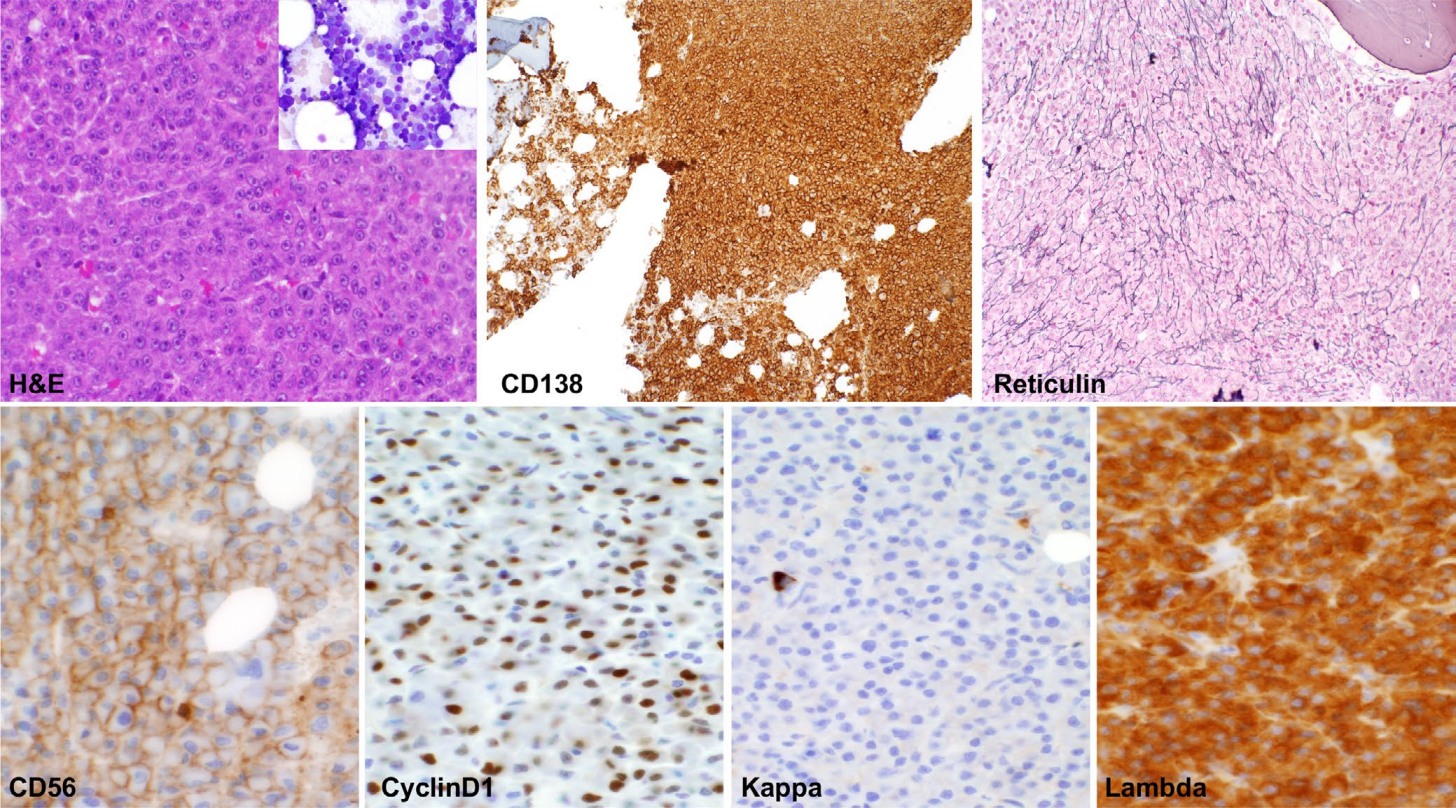
Standard H&E and immunohistochemical staining in bone marrow biopsy samples of patients with multiple myeloma. Bone marrow biopsy samples are routinely stained for H&E, CD138, Kappa and Lambda light chain, cyclinD1, CD56, and reticulin

Routine BM pathology evaluation for MM patients at Duke University Medical Center includes H&E staining, immunohistochemical (IHC) staining for CD138, CD56, cyclin D1, Kappa and lambda light chain, and Gomori stain for reticulin fibers (Figure 1). For reticulin staining, the slides were sectioned with a thickness of 4 microns and stained on an automated stainer. Trichrome staining was performed manually. Level of fibrosis was determined by pathologist review and scored according to the WHO: European consensus on grading bone marrow fibrosis and assessment of cellularity^26^ as: MF‐0 (absent) = no fibrosis; MF‐1 (mild) = low (fine reticulin network); MF‐2 (moderate) = intermediate (multifocal or diffuse non‐confluent fibrosis); or MF‐3 (severe) = high (marked and diffuse fibrosis) (Figure S1). BM cellularity and the percentage of CD138^+^ myeloma cells were extracted from the diagnostic pathology report.

### 2.4 Bone marrow biopsy JAK1/2 immunohistochemical staining

Archived BM paraffin blocks were cut into 5µM sections and fixed on slides. Microwave antigen retrieval was done in the presence of 1 mmol/L EDTA (pH 8.0) buffer. Slides were then incubated with anti‐CD138 (Cat# ms‐1793‐3, ThermoFisher, 1:20 dilution), anti‐JAK1 rabbit monoclonal antibody (#3344, Cell Signaling Technology, 1:100), or anti‐JAK2 antibody (#3230, Cell Signaling Technology, 1:50) for 30 minutes at room temperature, followed by Horseradish Peroxidase (HRP) labelled polymer antibody (DAKO, cat# K4001). Breast cancer tissue was used as the positive control for JAK1 and JAK2 staining and for optimization of antibody dilution and staining conditions (Figure S2).

### 2.5 Estimation of JAK2 immunohistochemical staining

**FIGURE 2:**
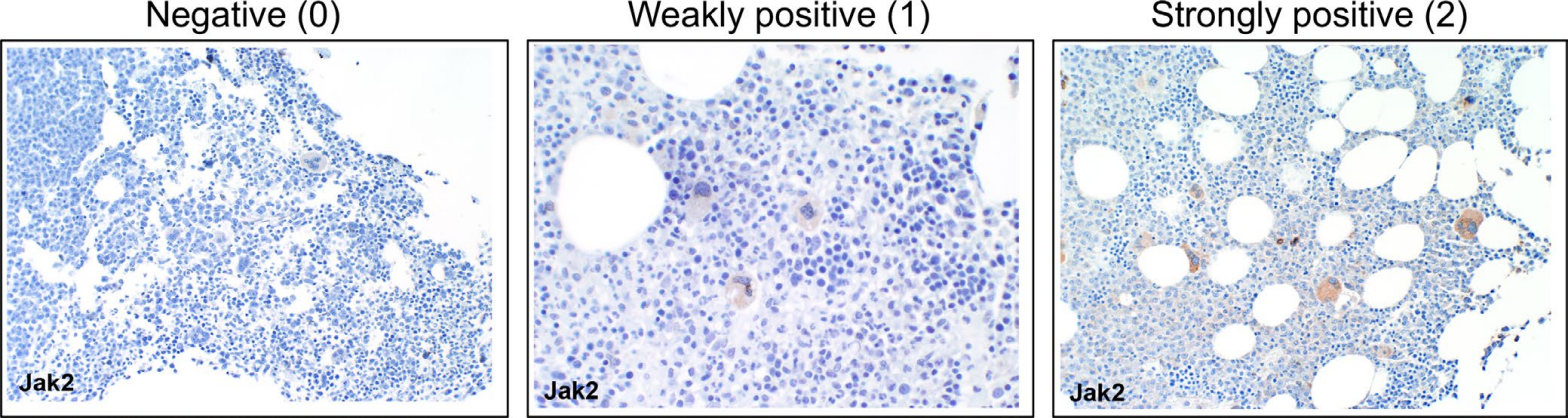
Classfication of JAK2 expression in megakaryocytes. Megakaryocyte JAK2 expression was classified as negative (0), weakly positive (1), or strongly positive (2)

Two measurements were obtained from JAK2 IHC staining: (a): the percentage of megakaryocytes expressing JAK2; and (b): the intensity of JAK2 expression. The percentage of megakaryocytes expressing JAK2 was calculated by dividing the megakaryocytes with positive JAK2 staining by the total megakayocytes, and were assigned a score ranging from 0 to 4 [score 0: no megakayocytes expressing JAK1/2; score 1:1%‐25% megakayocytes expressing JAK2; score 2:26%‐50% megakayocytes expressing JAK2; score 3:51%‐75% megakayocytes expressing JAK2; and score 4:76%‐100% megakayocytes expressing JAK2]. The intensity of expression was graded into: 0 = absent staining; 1 = weakly positive; or 2 = strongly positive (Figure 2). The IHC score for the overall expression level of JAK2 was represented as the sum of the score for percentage of expression and the score for the intensity of expression, and grouped as: low = IHC score of 0‐1; medium = IHC score of 2‐4; or high = IHC score of 5‐6. The percentage of JAK2 expression, the intensity of JAK2 expression and the overall IHC score were used separately to determine the correlation with bone marrow fibrosis, staging, cytogenetic stratification, treatment response, and survival etc

### 2.6 Statistical analysis

Summary patient characteristics as well as patient follow‐up were tabulated. Kaplan‐Meier estimation was used to determine median OS, as well as PFS, stratified by the presence and degree of BMF. The Log‐Rank test was used to test for differences in OS and PFS among patients with fibrosis versus those without. Differences in OS and PFS among patients with different degrees of fibrosis were also tested in this manner. Univariate logistic regression analysis was used to examine the associations between various clinical factors and presence of BMF. The association between JAK2 IHC score and various clinical characteristic and survival outcomes was examined using logistic regression (presence of BMF), ordinal logistic regression when appropriate (Cytogentics, cell differentiation, ISS staging, degree of fibrosis), as well as Cox‐Proportional Hazards modeling for survival outcomes (OS, PFS).

## 3 RESULTS

### 3.1 MM patients with BMF have a poorer prognosis even when treated with immunomodulatory agents and proteasome inhibitors

We determined the impact of presence of BMF on the clinical outcomes of NDMM patients in the current era of myeloma treatment. A total of 393 myeloma patients seen at Duke University Medical Center between 2003 and 2013 were included in the final analysis. The median follow‐up for living patients was 83 months (range: 3.9 to 212 months). A total of 314 (79.9%) patients were treated with an immunomodulatory agent (IMiD), 305 (77.6%) were treated with a proteasome inhibitor (PI), and 268 (68.2%) received both. Additionally, 213 (54.2%) patients went on to receive high dose chemotherapy followed by HSCT, and 96 (24.4%) patients received maintenance therapy (see Table S1 for the cohort's patient characteristics).

A total of 253 patients (64.4%) were evaluable for BMF. Of these, 122 (48.2%) had detectable BMF, while 131 (51.8%) had no BMF. Table 1 summarizes the clinical characteristics of patients with BMF and patients without BMF. Compared to patients without BMF, a higher number of patients in myeloma with BMF group received a bortezomib‐based regimen, and the presence of BMF was associated with higher ISS stages (Table 1). The degree of BMF was mild in 77 patients (63.1%), and moderate or severe in 43 patients (35.2%). The vast majority of BMF was reticulin fibrosis.

**TABLE 1:** Clinical characteristics of myeloma patients with or without bone marrow fibrosis

	Bone marrow fibrosis			*P*‐value*, #
	No	Yes	Total	
	(N = 131)	(N = 122)	(N = 253)	
Gender, n (%)				.9873*
Male	71 (54.2%)	66 (54.1%)	137 (54.2%)	
Female	60 (45.8%)	56 (45.9%)	116 (45.8%)	
Age at diagnosis				.5252#
N	130	121	251	
Mean (SD)	59.8 (10.21)	60.8 (10.55)	60.3 (10.37)	
Median	60.5	60.0	60.0	
Range	32.0, 84.0	35.0, 88.0	32.0, 88.0	
Race, n (%)				.5666*
Caucasian	85 (64.9%)	74 (61.2%)	159 (63.1%)	
African‐American	42 (32.1%)	45 (37.2%)	87 (34.5%)	
Other	4 (3.1%)	2 (1.7%)	6 (2.4%)	
Missing	0	1	1	
Year of diagnosis, n (%)				.6794*
2008 and prior	70 (53.8%)	62 (51.2%)	132 (52.6%)	
2009 to present	60 (46.2%)	59 (48.8%)	119 (47.4%)	
Missing	1	1	2	
Cytogenetic stratification, n (%)				.1859*
Standard	98 (86.0%)	83 (76.9%)	181 (81.5%)	
Intermediate	5 (4.4%)	10 (9.3%)	15 (6.8%)	
High risk	11 (9.6%)	15 (13.9%)	26 (11.7%)	
Missing	17	14	31	
M protein type, n (%)				.6257*
IgG	87 (71.3%)	75 (65.8%)	162 (68.6%)	
IgA	28 (23.0%)	30 (26.3%)	58 (24.6%)	
Other	7 (5.7%)	9 (7.9%)	16 (6.8%)	
Missing	9	8	17	
Light chain type, n (%)				.9177*
Kappa	84 (65.6%)	78 (65.0%)	162 (65.3%)	
Lambda	44 (34.4%)	42 (35.0%)	86 (34.7%)	
Missing	3	2	5	
ISS stage at diagnosis, n (%)				.0318*
1	34 (40.5%)	19 (22.1%)	53 (31.2%)	
2	21 (25.0%)	31 (36.0%)	52 (30.6%)	
3	29 (34.5%)	36 (41.9%)	65 (38.2%)	
Missing	47	36	83	
Chemotherapy regimen, n (%)				
Thalidomide‐based regimen	34 (26.0%)	25 (20.5%)	59 (23.3%)	.3046*
Lenalidomide‐based regimen	92 (70.2%)	95 (77.9%)	187 (73.9%)	.1667*
Pomalidomide‐based regimen	21 (16.0%)	27 (22.1%)	48 (19.0%)	.2162*, #
Bortezomib‐based regimen	94 (71.8%)	106 (86.9%)	200 (79.1%)	.0031*
Carfilzomib‐based regimen	18 (13.7%)	19 (15.6%)	37 (14.6%)	.6801*
Ixazomib‐based regimen	4 (3.1%)	2 (1.6%)	6 (2.4%)	.4601*
Elotuzumab, daratumumab or panobinostat – based regimen	8 (7.4%)	3 (3.0%)	11 (5.3%)	.1964*
Hematopoietic stem cell transplant type, n (%)				.1394*
No	63 (48.1%)	70 (57.4%)	133 (52.6%)	
Autologous HSCT	68 (51.9%)	52 (42.6%)	120 (47.4%)	
Allogeneic HSCT	2 (1.5%)	4 (3.3%)	6 (2.4%)	
Maintenance Therapy, n (%)				
Lenalidomide	22 (16.8%)	20 (16.4%)	42 (16.6%)	.9318*
Bortezomib	10 (7.6%)	10 (8.2%)	20 (7.9%)	.8683*

* Chi‐Square *P*‐value.

^#^ Kruskal‐Wallis *P*‐value.

**FIGURE 3:**
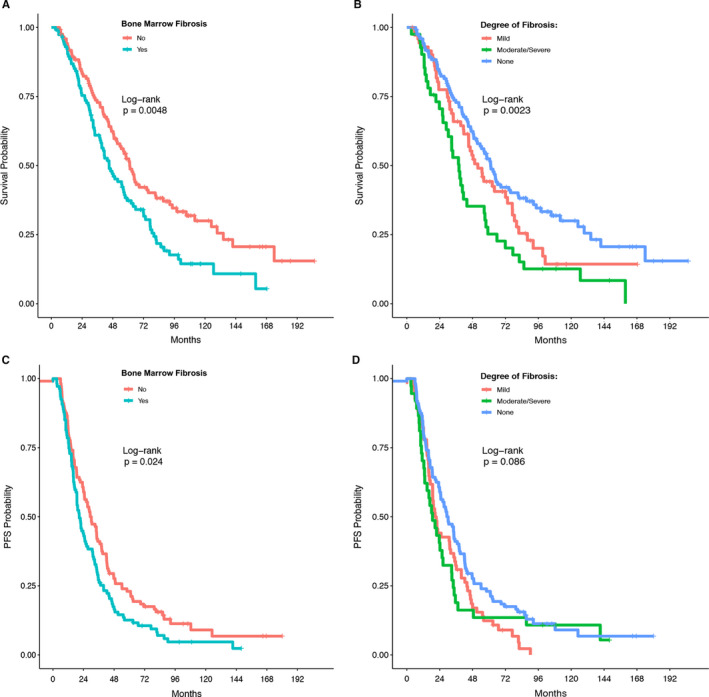
The association of bone marrow fibrosis with overall survival (OS) and progression‐free survival (PFS) in patients with newly diagnosed multiple myeloma. The Log‐Rank test was used to test for differences in OS and PFS among patients with fibrosis versus those without (A and C). Differences in OS and PFS among patients with different degrees of fibrosis were also tested in this manner (B and D). A and B: overall survival. C and D: progression‐free survival

We first determined the association between the presence of BMF and OS or PFS in our cohort of patients. The presence of BMF was associated with shorter OS. Median OS was 61.2 (95% CI: 51.5‐81.2) months in patients without BMF, and 45.1 (95% CI: 38.7‐57.0) months in patients with BMF (log‐rank *P* = .0048) (Figure 3A). Patients with moderate or severe BMF had a particularly poor prognosis with an OS of 38.1 (95% CI: 28.8‐57.0) months (*P* = .0023) (Figure 3B). Similarly, the presence of BMF was associated with poorer PFS with median PFS in patients without BMF of 30.2 (95% CI: 24.7‐38.0) months, compared to 21.1 (95% CI: 18.8‐27.5) months in patients with BMF present (log‐rank *P* = .024) (Figure 3C). Patients with moderate or severe BMF had a PFS of only 18.8 (95% CI: 13.1‐32.7) months (logrank *P* = .085) (Figure 3D).

Multivariate analyses were performed to determine whether BMF is an independent variate correlating with OS or PFS in myeloma patients (Table 2). After adjusting for age, race, cytogentic risk, and ISS stage at diagnosis, the presence of BMF was not an independent variate correlating with OS or PFS in myeloma patients.

**TABLE 2:** Multivariate analysis for OS and PFS in myeloma patients

	Overall survival (n = 143)			Progression‐free survival (n = 131)		
	HR	95% CI	*P*‐value	HR	95% CI	*P*‐value
BMF			.26			.12
No (ref.)	–	–		–	–	
Yes	1.27	0.84‐1.92		1.39	0.92‐2.10	
Age (1 y increase)	1.03	1.01‐1.06	**.0032** **	1.01	0.99‐1.03	.25
Race			**.02** *			.32
Caucasian (ref.)	–	–	–	–	–	–
African‐American	1.69	1.09‐2.63	**.02** *	1.32	0.87‐2.00	.19
Other	0.23	0.03‐1.72	.15	1.79	0.39‐8.23	.45
Cytogenetic			**.02** *			.29
Stratification						
Standard (ref.)	–	–	–	–	–	–
Intermediate	1.86	0.87‐4.00	.11	1.48	0.74‐2.96	.27
High Risk	2.06	1.17‐3.61	**.01** *	1.47	0.82‐2.64	.19
ISS Disease Stage			**.05** *			**<.001** ***
1 (ref.)	–	–	–	–	–	–
2	1.46	0.84‐2.51	.18	1.60	0.96‐2.66	.07
3	1.92	1.14‐3.25	**.01** *	2.77	1.68‐4.55	**<.001** ***

* *P* < .05.

^**^ *P* < .01.

^***^ *P* < .001.

### 3.2 The presence of bone marrow fibrosis is associated with higher myeloma ISS stage

We performed logistic regression analysis to determine the factors that are associated with presence of BMF. The factors we analyzed included: BM cellularity, the percentage of CD138^+^ myeloma cells by IHC staining, ISS stage, cytogenetics risk, and the M protein level (Table 3). Consistent with previous reports, the presence of BMF was associated with higher myeloma cell infiltration, as evidenced by increased BM cellularity and a higher percentage of myeloma cells. Furthermore, the occurrence of BMF correlated with ISS stage (*P* = .03), but had no correlation with the cytogenetic risk nor the M protein level (Table 3).

**TABLE 3:** Association of bone marrow fibrosis with various patient characteristics

Association with bone marrow fibrosis	OR	95% CI	*P*‐value
Bone marrow cellularity (%)	1.03	1.01‐1.04	<.0001
% bone marrow myeloma cells	1.02	1.01‐1.03	<.0001
ISS disease stage			.03
1 (ref.)	–	–	–
2	2.64	1.20‐5.81	.016
3	2.22	1.06‐4.68	.036
Cytogenetic stratification			.20
Standard (ref.)	–	–	–
Intermediate	2.36	0.78‐7.18	.29
High risk	1.61	0.70‐3.70	.92
Serum M protein level (g/dL)	1.01	0.89‐1.15	.87

To distinguish whether the poorer prognosis seen in the presence of BMF was due to the higher ISS stage in myeloma patients with BMF, we stratified patients by ISS stages and compared the OS and PFS of patients with or without BMF in each ISS stage. As shown in Table 4, after fixing ISS stage, the effects of BMF on OS and PFS were not significant anymore. This indicates that the significant effects of BMF on OS and PFS as shown in Figure 3 are likely due to a difference in ISS stage.

**TABLE 4:** Median OS and PFS in myeloma patients with or without BMF stratified by ISS stage

ISS	BMF status	Event/Total	Median OS (95% CI)	*P*‐value*	Event/Total	Median PFS (95% CI)	*P*‐value*
1	No (ref)	18/33	81.2 (61.1‐NE)		22/30	45.2 (28.6‐87.2)	
	Yes	13/18	72.1 (49.5‐126.7)	.09	14/16	35.8 (16.6‐87.3)	.13
2	No (ref)	13/18	43.4 (30.6‐NE)		16/18	29.2 (16.8‐86.2)	
	Yes	24/29	39.3 (29.4‐78.6)	.18	27/27	21.5 (18.9‐31.4)	.15
3	No (ref)	23/27	44.2 (35.8‐61.2)		21/22	15.1 (12.2‐38.0)	
	Yes	23/33	33.9 (27.5‐71.9)	.52	29/32	15.4 (10.8‐22.0)	.84

* log‐rank test.

### 3.3 JAK2 expression is detected predominantly in the megakaryocytes of bone marrow samples

The molecular mechanism(s) underlying the development of BMF in MM are not fully understood. The JAK signaling pathway is important in the survival and proliferation of myeloma cells as well as in the proinflammatory cytokine induced cell signaling pathway.^27^, ^28^ We thus determined the role of JAK expression in BMF of myeloma patients. To this end, we performed IHC staining for JAK1 and JAK2 protein expression on the archived BM biopsy samples of 99 NDMM patients (50 of whom had BMF, and 49 of whom did not have BMF).

For JAK1 IHC, we used the 6G4 rabbit anti‐JAK1 monoclonal antibody. This antibody was previously successfully used for IHC staining^29^ and stained positively in breast cancer tissues (Figure S2). For JAK2 IHC, the D2E12 rabbit anti‐JAK2 monoclonal antibody^29^, ^30^, ^31^ was used. Using the 6G4 anti‐JAK1 antibody no JAK1 expression was observed on the BM myeloma cells or on the BM microenvironment (data not shown). Out of 99 patients, only 1 (1.01%) patient had JAK2 expression on CD138 ^+ ^myeloma cells (Figure S3). JAK2 expression was observed predominanatly on megakaryocytes and to a less extent on the endothelial cells and the smooth muscles of the microvessel walls (Figure S3). We classified megakaryocyte JAK2 expression as negative (0), weakly positive (1) or strongly positive (2) (Figure 2). Of the 99 patients tested, 32 (32.3%) had no detectable JAK2 expression, 46 (46.5%) were weakly positive, and 21 (21.2%) were strongly positive. We also determined the percentage of megakaryocytes that expressed JAK2 in the samples that were either weakly or strongly positive for JAK2. The percentage of JAK2 ^+^ megakaryocytes ranged from 10% to 100% (Table 5).

**TABLE 5:** JAK1 and JAK2 expression in bone marrow biopsy samples of newly diagnosed multiple myeloma patients

	JAK1	JAK2, intensity n (%)	JAK2 ^+^ megakaryocytes, % (mean, Sd, range)
Plasma cells	–	1 (1.01%)	
Bone marrow microenvironment	–	+	
Megakaryocyte	–	+	
Negative (0)		32 (32.3%)	0,0
Weak positive (1)		46 (46.5%)	63.3, 22.5 (10‐100)
Positive (2)		21 (21.2%)	75.2, 18.1 (30‐100)
Vascular smooth muscle	–	+	
Endothelium	–	+	

### 3.4 The expression of JAK2 in megakaryocytes correlates with myeloma ISS stages

We examined the correlation of JAK2 expression in BM megakaryocytes with clinical outcomes of myeloma patients. The percentage of JAK2 expression, the intensity of JAK2 expression and the overall IHC score were used separately to determine the correlation with BMF, myeloma ISS stages, cytogenetic stratification, treatment response, and survival etc. The results were similar with using the percentage of JAK2 expression, the intensity of JAK2 expression or the overall IHC score.

**FIGURE 4:**
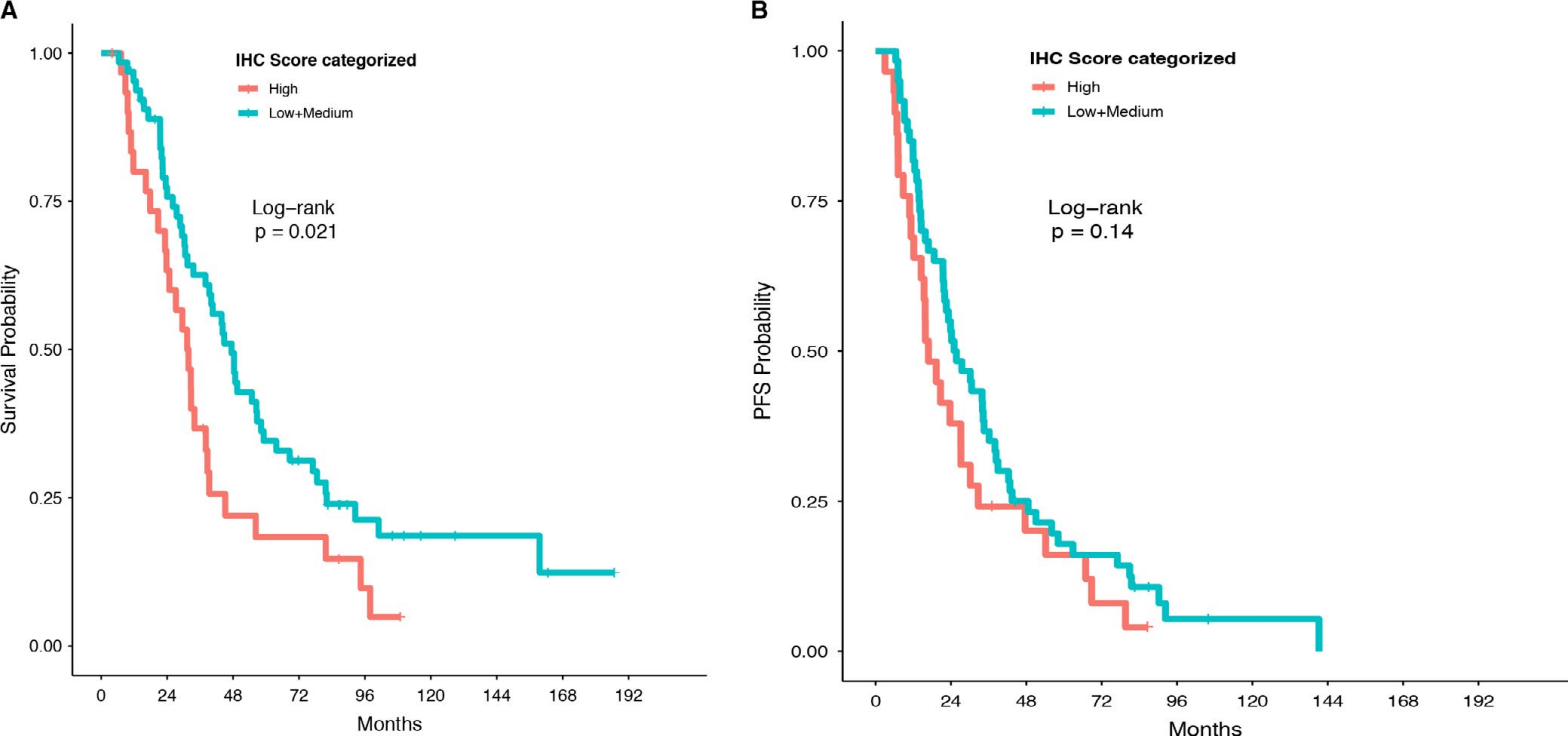
The association of bone marrow megakaryocyte JAK2 expression with overall survival (OS) and progression‐free survival (PFS) in patients with newly diagnosed multiple myeloma. The JAK2 IHC score was the sum of the score for percentage of expression and the score for the intensity of expression, and categorized as: low = IHC score of 0‐1; medium = IHC score of 2‐4; or high = IHC score of 5‐6. A: overall survival. B: progression‐free survival

We used the JAK2 IHC score that incorporated both the intensity and the percentage of JAK2 expression and was the sum of the score for percentage of expression and the score for the intensity of expression. Compared to myeloma patients with low and medium JAK2 IHC score, patients with high JAK2 IHC score showed a signficantly worse OS and a trend for worse PFS (Figure 4A,B).

We performed logistic regression analyses to determine the patient characteristics that are associated with JAK2 expression. JAK2 expression did not correlate with the presence or the degree of BMF, nor the cytogenetics or myeloma cell differentiation, but was significantly associated with ISS stage (Table 6).

**TABLE 6:** Association of JAK2 IHC score with various patient characteristics

Outcome	OR (+1 increase in JAK2 IHC score)	95% CI	*P*‐value
Bone marrow fibrosis Yes vs No	0.97	0.81‐1.15	.72
Degree of bone marrow fibrosis Mild/moderate vs none Moderate/severe vs none/mild	1.03	0.88‐1.22	.69
Cytogenetics Intermediate/high risk vs standard High risk vs standard/intermediate	1.00	0.80‐1.25	.99
Cell differentiation Moderate/well vs poor Well vs moderate/poor	0.87	0.74‐1.03	.11
ISS stage 2/3 vs 1 3 vs 1/2	1.33	1.08‐1.64	**.0071**

Bold values indicates *P* < .01.

## 4 DISCUSSION

In this study, we determined the impact of BMF on the clinical outcomes of NDMM patients in the current era of myeloma treatment. Our study represents the largest dataset (393 patients) with the longest follow up (83 months) reported so far examining the impact of BMF in MM. Moreover the majority of our patients were treated with IMiDs and/or PIs and went on to receive autologous HSCT, the current standard practice for NDMM. We found that BMF is common in NDMM and occurs in 48.2% patients that were evaluated for BMF. We further showed that BMF correlates with myeloma ISS stage. Importantly, our study demonstrated that even in an era where newer therapies have significantly improved outcomes for MM patients, the presence of BMF still negatively impacts the outcomes of patients with NDMM indirectly, likely by associating with higher ISS stage. These data emphasize the importance of determining the presence and degree of BMF at time of MM diagnosis, and suggest a role for adjunctive therapies that target BMF in MM patients with co‐existing BMF.

An increase in BM fibrous tissue has been observed in both malignant and nonmalignant hematologic diseases where there is rapid proliferation of marrow cells.^32^ Previous studies suggested that the degree of BMF was related to the magnitude of plasma cell infiltration.^17^, ^18^ Consistent with these findings, our current study demonstrated that increased BM cellularity and higher percentage of myeloma cell involvement are associated with the occurrence of BMF (Table 3). We have also noted that the BM fibrosis occurs in close proximity to the myeloma cell clusters (Figure S4), suggesting that myeloma cells directly (by cell/cell contact) or indirectly (through secretion of cytokines, chemokines or other factors) affect the formation of BMF.

In our univariate analysis, BMF was found to be significantly correlated with OS and PFS in patients with MM, but this effect was not maintained in the multivariate analysis after adjusting for other baseline characteristics. One of the possibilities is that the study is underpowered and with a larger sample size and more events, BMF may have independent prognostic value. We have calculated the power for the effect of BMF on PFS and OS in the multivariate analysis to be 80% if the sample size is around 600, but it needs to be over 1000 for OS. The other possibility is that BMF indirectly affects PFS and OS of myeloma patients through its association with ISS stage. This was consistent with our finding that after fixing the ISS stage, BMF does not have an effect on PFS or OS (Table 4). Thus, by association with worse ISS stage, BMF affects PFS and OS of myeloma patients indirectly. The effect of BMF on PFS and OS may be similar to that seen with age, lactate dehydrogenase, hemoglobin or the percentage of bone marrow plasma cells which when analyzed in 10,750 NDMM patients were noted to be correlated with OS on univariate analysis, but were not independent factors in multivariate analysis.^2^

The JAK/STAT pathway is a central pathogenic component in myelofibrosis seen in myeloproliferative neoplasm (MPN)^33^ and is largely due to the JAK2 V617F mutation, calreticulin mutation or c‐MPL mutation. Several lines of evidence suggest that mutation of JAK2 V617F is absent in MM and thus does not play a role in the pathogenesis of BMF.^34^, ^35^ However, using PCR technology it was found that 57% of patients overexpressed JAK2 and 27% overexpressed JAK1.^36^ We performed JAK1 and JAK2 IHC staining on archived BM biopsy samples of 99 NDMM patients. For all the 99 myeloma cases, JAK1 was negative regardless of neoplastic and mesenchymal cells. The absence of positive JAK1 staining in all our patients could be due to: a) the absence or very low level of JAK1 expression in the BM or b) the non‐reactivity of the antibody we used for the IHC. The antibody we used for JAK1 staining (6G4 rabbit anti‐JAK1 monoclonal antibody) was used successfully for IHC in positive control tissues lessening the likelihood that the antibody was non‐reactive; however, additional staining with other antibodies specific to JAK1 is warranted to confirm our findings.

JAK2 was also negative on neoplastic cells except for one case, but showed positive staining in both endothelial and smooth muscle cells. Interestingly, we noted cytoplasmic staining in the BM megakaryocytes of 67 (67.7%) of our patient samples. Megakaryocytes have been reported to be associated with reticulin formation in autoimmune diseases and in some cancers.^37^, ^38^ Megakaryocytic hyperplasia is characteristic of MPNs, and it is believed that these cells may stimulate the fibrotic progression.^39^, ^40^ JAK2 inhibitors were found to help patients regardless of their mutation status.^27^, ^41^ Given that expression of JAK2 was located on nonneoplastic cells in our MM cases, it is possible that treatment with a JAK2 inhibitor could suppress BM megakaryocytes and result in subsequent downregulation of certain cytokines such as IL‐6, thus inhibiting the proliferation of myeloma cells as well as the fibrosis.^27^, ^42^, ^43^

## 5 CONCLUSIONS

BMF is common in NDMM, and MM patients with BMF (particularly those with extensive BMF) have a poorer prognosis even when treated with IMiDs and PIs. JAK2 expression was detectable in the majority of MM patients and correlated with myeloma disease stage. Our data emphasize the importance of determining the presence and degree of BMF at time of MM diagnosis, and suggest a role for adjunctive therapies that target BMF in MM patients with co‐existing BMF.

## CONFLICT OF INTERESTS

Yubin Kang received research funding from InCyte Corporation and Consultancy fee from Takeda Oncology USA. All other authors declare no competing conflicts of interest.

## AUTHORS’ CONTRIBUTIONS

BP performed the chart review, analyzed the data, and wrote the paper. YZ performed the pathology review and IHC measurement, and wrote the paper. GL performed the chart review and the research study. DF and PM designed the RedCap database and performed the research study. IB and ZL performed the statistical analyses. MD assisted in the chart review and database setup. LR provided valuable input on research design and data interpretation. EW assisted in pathology review, IHC measurement, and research design. YK designed the study, performed the chart review, and wrote the paper.

## Supporting information

Supplementary Material

## Data Availability

The data that support the findings of this study are available on request from the corresponding author. The data are not publicly available due to privacy or ethical restrictions.
