# Supplementary material for: The impact of bone marrow fibrosis and JAK2 expression on clinical outcomes in patients with newly diagnosed multiple myeloma treated with immunomodulatory agents and/or proteasome inhibitors
Source: Cancer Med. 2020 Jul 6;9(16):5869–80. doi: 10.1002/cam4.3265 (PMC7433821; doi:10.1002/cam4.3265)
Supplement: Supplementary file 1 — Supplementary Material [file CAM4-9-5869-s001.docx]

The impact of bone marrow fibrosis and JAK2 expression on clinical outcomes in patients with newly diagnosed multiple myeloma treated with immunomodulatory agents and/or proteasome inhibitors.

Supplementary materials

**Supplementary Table 1: patient characteristics**

| **Gender** | Male: 217 (55.2%)  Female: 176 (44.8%) |  |
| --- | --- | --- |
| **Race** | Caucasian: 247 (62.8%)  African American: 128 (32.6%)  Asian: 3 (0.8%)  Hispanic: 1 (0.3%)  Native American: 8 (2.0%)  Other: 4 (1.0%)  Unknown/missing: 2 (0.5%) |  |
| **Vital status** | Dead: 246 (62.6%)  Alive: 115 (29.3%)  Unknown: 32 (8.1%) |  |
| **Cytogenetics** | Standard: 246 (62.6%)  Intermediate: 20 (5.1%)  High risk: 37 (9.4%)  Unknown/missing: 90 (22.9%) |  |
| **M protein type** | IgG: 244 (62.1%)  IgA: 84 (21.4%)  IgM: 3 (0.8%)  IgD: 2 (0.5%)  IgE: 0 (0%)  Other (light chain): 21 (5.3%)  Unknown/missing: 41 (10.4%) |  |
| **Light chain type** | Kappa: 241 (61.3%)  Lambda: 127 (32.3%)  Unknown: 25 (6.4%) |  |
| **ISS stage** | I: 74 (18.8%)  II: 84 (21.4%)  III: 84 (21.4%)  Unknown: 151 (38.4%) |  |
| **Hematopoietic stem cell transplant** | Yes: 213 (54.2%)  No: 112 (28.5%)  Unknown/missing: 68 (17.3%) |  |
| **IMiD treatment** | Yes: 314 (79.9%)  No: 50 (12.7%)  Unknown/missing: 29 (7.4%) |  |
| **Proteasome inhibitor treatment** | Yes: 305 (77.6%)  No: 59 (15.0%)  Unknown/missing: 29 (7.4%) |  |

Supplementary Figure S1:

**Figure S1: Grading of bone marrow fibrosis.** Level of fibrosis was determined by pathologist review and scored as: absent = no fibrosis; mild = low (fine reticulin network); moderate = intermediate (multifocal or diffuse non-confluent fibrosis); or severe = high (marked, diffuse fibrosis, with or without areas of collagenization).


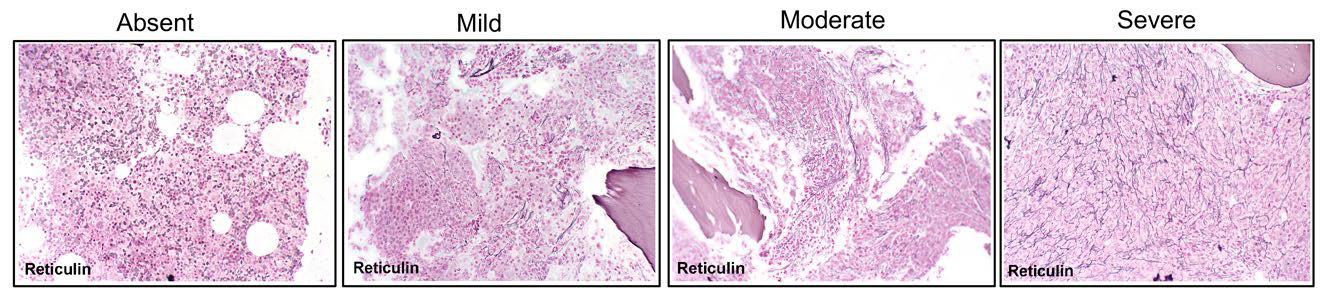


Supplementary Figure S2:

**Figure S2: Positive JAK1 and JAK2 staining in breast cancer tissues.**

**
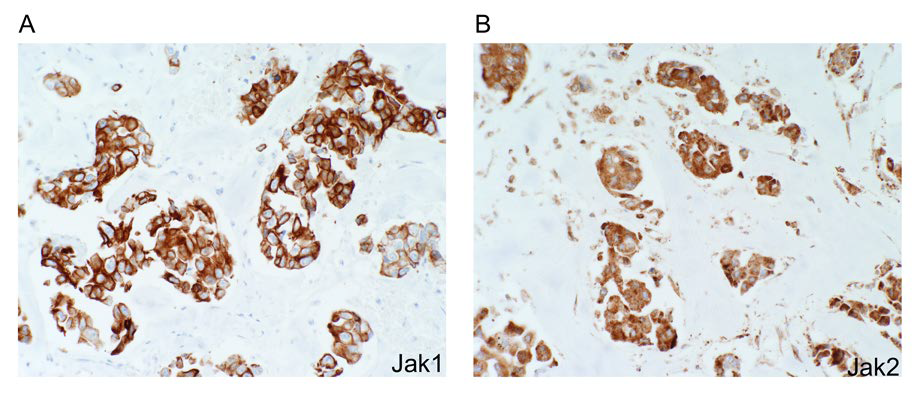
**

Supplementary Figure S3:

**Figure S3: Case with unequivocal expression of JAK2 protein both in myeloma cells and megakaryocytes.**


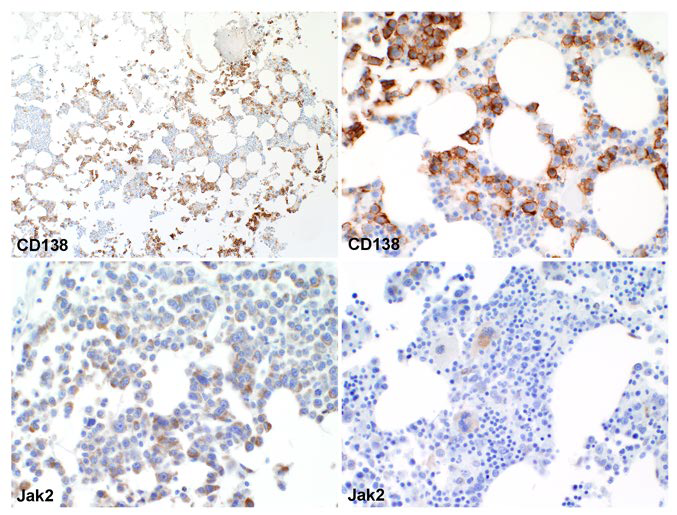


Supplementary Figure S4:

**Figure S4: Close proximity of bone marrow fibrosis to the myeloma cell clusters**

**
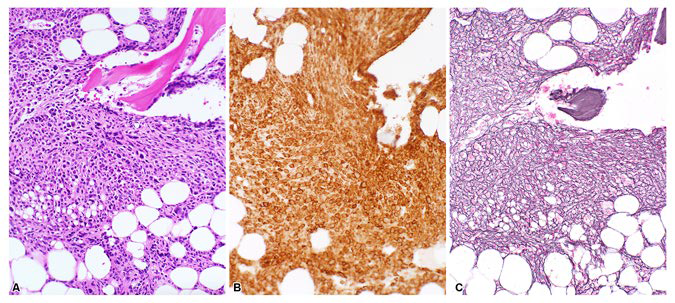
**
